# Interventions to promote healthy environments in family child care homes in Oklahoma—Happy Healthy Homes: study protocol for a randomized controlled trial

**DOI:** 10.1186/s13063-019-3616-9

**Published:** 2019-08-30

**Authors:** Susan B. Sisson, Alicia L. Salvatore, Deana Hildebrand, Tiffany Poe, Cady Merchant, Megan Slawinski, Chelsea L. Kracht, Julie A. Stoner, Naneida Alcala Lazarte, Lu Ann Faulkner Schneider, Jennifer Weber, Felecia Jones, Dianne Ward

**Affiliations:** 1 Behavioral Nutrition and Physical Activity Laboratory, Department of Nutritional Sciences, College of Allied Health, University of Oklahoma Health Sciences Center, 1200 N Stonewall Ave, AHB 3057, Oklahoma City, OK 73117-1215 USA; 2 Hudson College of Public Health, University of Oklahoma Health Sciences Center, 801 NE 13th Street, Oklahoma City, OK 73104 USA; 3 Christiana Care Health System, 4755 Ogletown-Stanton Road, Newark, Delaware 19718 USA; 4 Department of Nutritional Sciences, Oklahoma State University, Oklahoma City, USA; 5 School of Hospitality and Tourism Management, Oklahoma State University, Stillwater, USA; 6 Department of Health Promotion Sciences, College of Public Health, University of Oklahoma Health Sciences Center, Oklahoma City, USA; 7 Department of Biostatistics and Epidemiology, College of Public Health, University of Oklahoma Health Sciences Center, Oklahoma City, USA; 8 Division of Research and Data Analysis, State Department of Education, Oklahoma City, USA; 9 Department of Child Care Services, Oklahoma Department of Human Services, Oklahoma City, USA; 10 Division of Child Nutrition, Oklahoma State Department of Education, Oklahoma City, USA; 11 Mrs. Felecia’s Playhouse Preschool, Oklahoma City, USA; 12 Gillings School of Global Public Health, University of North Carolina Chapel Hill, Chapel Hill, USA

**Keywords:** Early care and education, Child care, Nutrition, Dietary intake, Environmental health, Pesticides, Intervention, Green cleaning, Implementation science, Obesity prevention

## Abstract

**Background:**

Early childhood is a critical period of development. Caregivers, including providers of early care and education (ECE), have a substantial influence on the health of young children. Family child care homes (FCCHs), which are small, licensed ECE businesses operated out of the residences of providers, are important settings for promoting child health. However, to date, few interventions to promote the health of children have been developed for FCCHs. The purpose of this article is to describe the protocol for Happy Healthy Homes, a pilot interdisciplinary, community-based study to improve FCCH environments and the health of children in Oklahoma. We describe the development and evaluation of two interventions to be tested in a matched attention randomized controlled trial: 1) a nutrition intervention aimed at enhancing the nutritional quality of meals served to young children, incorporating the Child and Adult Care Food Program best practices, and improving nutritional self-efficacy of providers; and 2) an environmental intervention aimed at increasing providers’ environmental health literacy, self-efficacy for integrated pest management (IPM), and awareness of less toxic cleaning practices and FCCH provider cleaning behaviors.

**Methods:**

Both interventions are informed by common theoretical principles and are matched in attention (i.e., 6 h), format (i.e., two individual 90-min educational home visits and a 3-h small group class) and materials (i.e., tool kit of educational materials and supplies tailored to the allocated intervention). A randomized trial of both interventions is currently underway with 52 FCCH providers in the Oklahoma City metropolitan area who participate in the Child and Adult Care Food Program. Observed and self-reported measures will be collected at baseline, and 3 months and 12 months after baseline measurements. Randomization to one of the two interventions will occur after baseline data collection.

**Discussion:**

This study aims to support FCCH providers in creating healthier FCCH environments for nutrition and environmental health. Successful completion will provide critical information about the nutritional quality and the environmental health of children in FCCHs, as well as much needed evidence about the efficacy of two community-based interventions to improve the nutrition and environmental health of children in home-based ECE settings.

**Trial registration:**

Clinicaltrials.gov, NCT03560050. Retrospectively registered on 23 May 2018.

**Electronic supplementary material:**

The online version of this article (10.1186/s13063-019-3616-9) contains supplementary material, which is available to authorized users.

## Background

Early childhood is a critical developmental period and one that largely determines health throughout the life course [1]. This is particularly true for neurological development, where the most substantial gains in development occur within the first 1000 days of life [2]. Early care and education (ECE) programs are of critical importance for addressing social and environmental determinants of child health [3]. In the United States, children with employed mothers spend an average of 32 h per week in care [4], and approximately 60% of 3- to 5-year-old children are in nonparental care [5]. As such, the social and physical environments of ECEs may have substantial influences on the health of children [6], and ECE providers are in a strategic position to positively influence the health behaviors of children [7, 8]. Interventions to enhance the quality of ECE for the health of children have been prioritized by multiple agencies, including the White House Childhood Obesity Task Force [9], the National Academy of Medicine (NAM; formerly known as the Institute of Medicine) [10], the Children’s Environmental Health Network [11], the National Association of County and City Health Officials [12], the American Academy of Pediatrics [13], the American Public Health Association [14], and the National Resource Center for Health and Safety in Child Care and Early Education [15].

School-based health interventions have long received interest, but relatively few interventions have focused on ECE environments. Those interventions that have included ECE have predominantly focused on head start and center-based care [16]. Few interventions have focused on family child care homes (FCCHs), even though 26% of all ECE attendance is in FCCHs [17]. FCCHs are small, licensed ECEs in the homes of providers that care for up to 12 children [18]. Given the unique situation of operating a business that is subject to licensure and regulations and operated out of a personal home with few administrative personnel and resources, FCCH providers need interventions that will support them in creating healthier environments for young children. For instance, the NAM expressed the urgent need to understand the obesity-related attributes, specifically nutrition, of the unique environment of FCCHs [19]. Similarly, the American Public Health Association’s Comprehensive Framework for Protecting Children’s Environmental Health urged state and local child care licensing officials to adopt environmental health standards included in the third edition of Caring for Our Children [13] as requirements for licensing [14].

In terms of health, the food and nutrition environment has been prioritized for intervention in ECE and FCCHs [9, 19, 20]. ECE providers serving low-income children can participate in the Child and Adult Care Food Program (CACFP), which reimburses qualifying food costs [21]. CACFP participation is associated with increased access to nutritious foods [22, 23]. However, there are variations in the fidelity with which it is implemented [24]. These variations may compromise overall nutritional quality for some children [24, 25] and leave substantial room for improvement. To date, policies and interventions to improve nutrition in FCCH remain minimal [26].

Similarly, reducing the exposure of children to household chemicals in ECE and FCCH environments has been recognized as an important area of intervention [13, 27–32]. These chemicals are ubiquitous and commonly found in cleaning products, pest control products, and consumer products, such as plastics and toys [33]. Because of their smaller size, immature metabolic systems, exploratory behavior, extended periods spent on the floor and ground, and rapid physical and neurologic development and growth, children are not only more likely to experience disproportionate chemical exposures but also are more vulnerable to associated health effects [34]. Studies indicate that ECE environments contain a wide array of household chemicals and other environmental toxins [35–40]. Evidence of, and growing concerns about, the negative health impacts of environmental exposures, including increased risks of asthma and respiratory disease, endocrine disruption, childhood cancers, and behavioral and neurodevelopmental disorders, has led health professionals and children’s health advocates to highlight ECE environments as critical settings for reducing exposures and protecting children’s health [40–42]. While there are few policies and interventions to reduce potentially hazardous environmental exposures to children in FCCHs, the interventions conducted to date with ECE centers [43–45] and FCCHs [46, 47] have been promising.

Interprofessional team science and randomized study designs offer opportunities for developing and testing much needed interventions for FCCHs that address multiple determinants of health and protect and promote child health across multiple spectrums of opportunity. Accordingly, community-engaged research has the opportunity to develop and integrate more valued and sustainable health interventions [48, 49]. In this article, we describe Happy Healthy Homes (hereafter referred to as Happy), an interdisciplinary, community-based study in Oklahoma to improve FCCH environments and children’s health through the development and testing of two matched interventions, a nutritional intervention and an environmental health intervention, among FCCH providers (*n* = 52) in the Oklahoma City metropolitan area.

## Methods

### Study design

A randomized, matched-attention, controlled intervention trial will be used to evaluate the two interventions among a cohort of FCCH providers. Observed and self-report measures will be collected at participating FCCHs at three time points: at baseline, and 3 months and 12 months after baseline. All data will be collected at the homes of providers on two unannounced dates. After baseline data collection, providers will be randomly assigned to the nutrition intervention or to the environmental health intervention, both comprised of two in-home 90-min individual education sessions and a 3-h small group class with 4–6 participants (i.e., approximately 6 h of intervention completed over 3 months). Providers who complete the interventions will earn six free, prequalified continuing education credits (i.e., half the annual requirement [18]) and research incentives for participation in measurement. Additionally, participants will receive approximately $250 in toolkit supplies over the course of intervention participation. This study is registered with Clinicaltrials.gov (NCT03560050). See Additional file 1 for the SPIRIT checklist, and Additional file 2 (Appendix A) for the World Health Organization Trial Registration Data. This is protocol version 1, finalized October 2017.

### Study population

FCCH providers (small, home-based ECE providers [18]) who participate in the CACFP and are located within the Oklahoma City metropolitan area (approximately a 60-mile radius) within the United States will be eligible to participate in the Happy study if they plan to remain in business for at least 12 months. In Oklahoma, there are nearly 2000 FCCH providers [50], 88% of which participate in the CACFP [51]. In the Oklahoma City area, there are nearly 160 FCCH providers participating in the CACFP [52].

### Recruitment

Recruitment will occur between October 2017 and November 2018 for three waves/cohorts of participants and is guided by other FCCH health interventions [53]. To participate in the CACFP and receive meal reimbursement funds, FCCH providers must work with a sponsoring organization. We established partnerships with the three sponsoring organizations that serve the Oklahoma City area FCCH providers. We will employ passive and active recruitment approaches to enroll participants, including brief presentations about the study, distribution of recruitment materials at sponsor-required annual trainings, and recruitment calls to FCCH provider membership lists provided by sponsor organizations. Interested FCCH providers will be screened for eligibility by trained graduate research assistants on the telephone or in person, and will be enrolled upon successful screening and informed consent. Consent (Additional file 2: Appendix B) will be obtained at the first in-person visit with research personnel for collecting baseline measures. Retention efforts include provision of a certificate of completion, follow-up quality-control telephone calls, and payment for subsequent measurement visits even if the intervention was not completed. All study records are confidential and are not shared outside the immediate research team, including to sponsoring organizations.

### Randomization

**Fig. 1:**
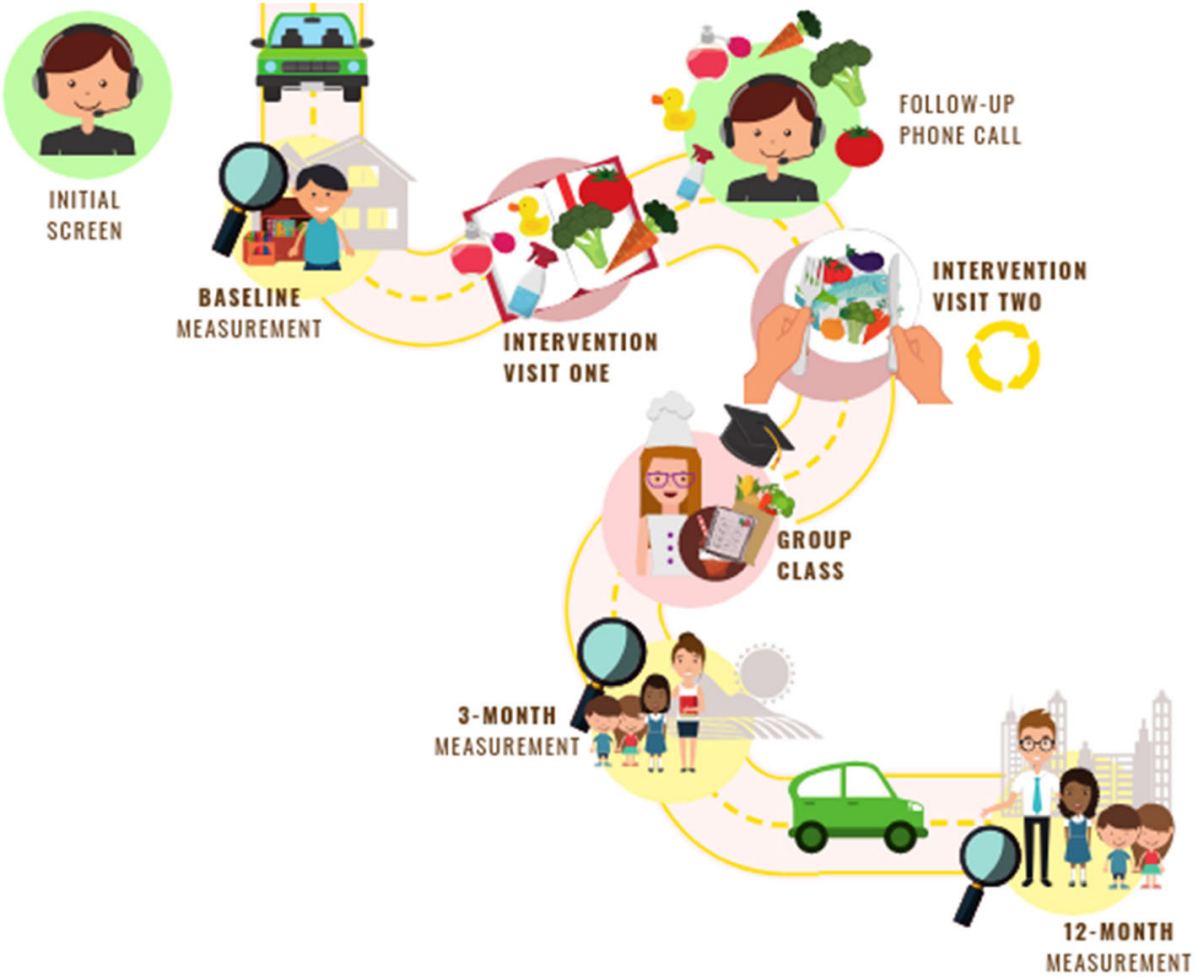
Happy Healthy Homes intervention trial time-course, indicating screening, measures timing, and intervention components

**Fig. 2:**
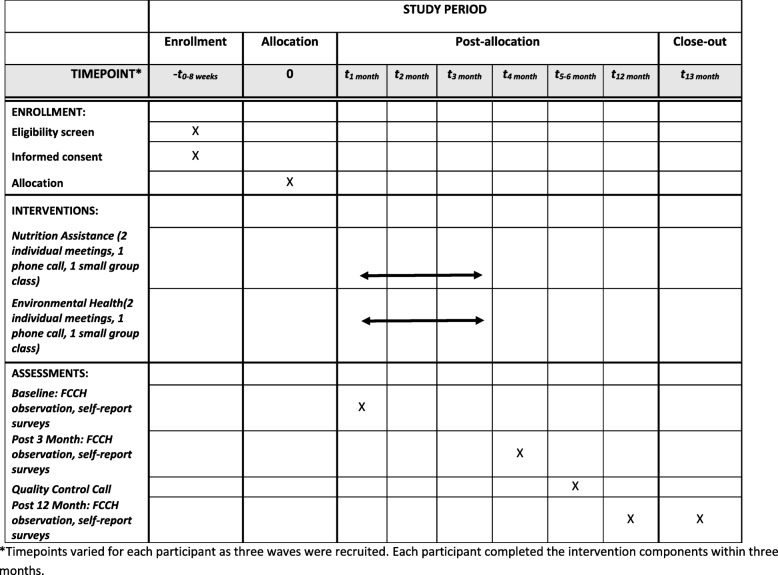
SPIRIT figure. Schedule of enrollment, interventions, and assessments for each wave. *Timepoints varied for each participant as three waves were recruited. Each participant completed the intervention components within 3 months. FCCH family child care home

Of the 52 providers to be enrolled in the Happy intervention trial, 26 will be randomly allocated to each intervention arm. Figure 1 shows the protocol time course for study participants. Figure 2 shows the schedule of enrollment, interventions, and assessments for each of the three waves. Upon enrollment, participants will be randomly assigned to one of the two study interventions by the study biostatistician using R software (www.r-project.org) and a randomization sequence generated in randomly chosen blocks of size four or six. Measurement staff will be blinded to the treatment group until after baseline measures have been collected.

### Intervention development

With input from our Study Advisory Committee, comprised of study investigators and partners from FCCH providers, Oklahoma State University, Cooperative Extension, Department of Human Services Division of Child Care Licensing, Center for Early Childhood Professional Development, and the Department of Education Division of Child Nutrition, as well as a review of relevant literature and intervention curricula, we developed two theory-based pilot interventions for FCCH providers. The nutrition pilot intervention aims to enhance the nutritional quality of meals served to young children, incorporate the CACFP best practices, and to increase the self-efficacy of providers for nutrition-related behaviors. The environmental health pilot intervention aims to increase providers’ environmental health literacy, self-efficacy for integrated pest management (IPM), awareness of less toxic cleaning, and providers’ use of less toxic cleaning practices. As mentioned previously, the two interventions are informed by common theoretical principles and matched in attention (i.e., 6 h), format (i.e., two individual 90-min educational home visits and a 3-h small group class), and materials (i.e., tool kit of educational materials and supplies tailored to the allocated intervention). Similarly, the two interventions are designed to meet a variety of educational competencies (Oklahoma Core Competencies [54], Child Development Associate Content Areas [55] and Quality Rating Improvement System Reaching for the Stars Criteria [56]). The development of the interventions took place from May to October 2017. Modules were piloted with an FCCH provider on the study advisory committee who was not included in data collection; recommended and identified changes were incorporated into both interventions after piloting.

### Trial oversight

The Study Advisory Committee, described in the previous section, meets biannually for reporting and discussion on trial progress and to address any issues with recruitment, retention, contamination, and so on. Meetings are held in person and distance options (i.e., telephone or Zoom) are provided. The committee also receives regular reports on study progress. Human subject and ethical oversight is provided by the University Institutional Review Board.

### Theoretical foundations

The theoretical foundations for the Happy study interventions build on the existing evidence base for public health promotion interventions in ECE [57]. Specific theoretical constructs and parallel intervention activities are summarized in Table 1. Our study is informed by the Social Ecological Model [58–64], which demonstrates the complex and dynamic inter-relationships between individual and environmental factors that influence health and health behavior. As operationalized in this study, the innermost circle represents the child, the institutional level is the FCCH, and the policy influence is federal and state licensure and food program (CACFP) regulations. Thus, the regulations influence the child via the FCCH. By focusing on outer circles of policy, health behavior change can reach a greater number of children in a more sustainable manner. In particular, this study targets influencing the knowledge, beliefs, skills, and attitudes of the FCCH providers.

**Table 1:** Theoretical constructs and intervention activities of Happy interventions

Theoretical constructs	Intervention activities
Social cognitive theory	
Behavioral capability	Educational lessons, hands-on activities, cooking class, making household cleaners
Self-control	Goal setting, problem solving, goal progress evaluation
Expectancies (value of outcome)	Educational lessons integrated with qualitative teacher self-perspectives
Observational learning	Hands-on activities, cooking class, making household cleaners, community partner involvement
Self-determination theory	
Proactive	Elective modules, hands-on activities
Personal importance	Educational lessons integrated with qualitative teacher self-perspectives
Interest	Hands-on activities
Adult learning principles	
Active learning	Elective modules, hands-on activities
Preconceptions	Reflective listening
Understanding	Educational lessons include “why”
Self-assessment	Goal setting, progress check-ins, and troubleshooting
Community-centered	Small-group cooking and children’s environmental health classes
Social support	
Instrumental support	Hands-on activities, cooking class, toolkit materials
Informational support	Educational lessons, goal setting, troubleshooting
Appraisal support	Discussion and troubleshooting of specific, measurable, attainable, realistic, and time-sensitive (SMART) goal progress and challenges experienced
Peer support	Small-group cooking and children’s environmental health classes with other providers

Our interventions are also informed by social cognitive theory [65, 66], social support [67], self-determination theory [68], and adult learning practices [69]. We operationalize five constructs of the social cognitive theory: 1) behavioral capability; 2) expectancies; 3) self-control; 4) observational learning; and 5) self-efficacy [65, 66, 70]. We also employ various types of social support, including instrumental, informational, and appraisal support to participants from interventionists; during the small group class, providers build mutual peer support and experience peer-to-peer learning [67, 70]. We incorporate self-determination theory via proactive, provider-directed goal setting, and facilitate providers’ participation in hands-on and participatory activities to build competence and interest [68, 70]. Consistent with self-determination theory [71] and adult learning practices [72], providers set their own goals and select the modules for the second educational session. Additional aspects of adult learning practices are integrated in the interventions through the community-centered group class, active learning experiences, discussion of preconceptions, and ongoing facilitation of participants’ understanding and self-assessment [72].

### Intervention modules

As mentioned previously, the intervention curricula that we created for both interventions are comprised of individual and group educational training sessions. A list of modules is provided in Tables 2 and 3. The curricula are designed to be delivered over a 3-month period and, other than the check-in telephone call completed between the first and second sessions, are delivered in person. In-person encounters were selected to establish stronger connections with participants and to address the interest in adult interactions of FCCH providers [73]. Similarly, interventionists work with the same participants throughout the study for consistency and relationship building.

**Table 2:** Happy nutrition intervention core and elective modules and provider learning objectives

Modules	Provider learning objectives
Core modules	
SMART goals	• Describe specific, measurable, attainable, relevant, and timely (SMART) goals • Practice writing a SMART goal • Develop a SMART goal for the family child care home (FCCH) for each subsequent module
Why met best practices	• Identify Child and Adult Care Food Program (CACFP) best practices and their importance for nutrient intake • Identify barriers and possible solutions to meeting CACFP best practices
Portion distortion: what is the right size?	• Recognize age-appropriate portion sizes • Identify appropriate preschool portion sizes
Staff behaviors: leading the way for healthy eating	• Understand positive role modeling for child feeding behaviors
Elective modules	
A fluid situation	• Understand differences in juice and whole fruit and importance of milk and water • Identify different ways to serve fruit and increase water intake
Begin with breakfast	• Recognize importance of breakfast for child wellness • Learn alternative breakfast ideas to meet best practices • Identify solutions to common breakfast challenges
Cooking across the rainbow	• Recognize importance of having a colorful plate • Learn nutrients associated with different colors • Describe ways to include more colorful fruit and vegetables in child meals
Getting children in the kitchen	• Recognize benefits of cooking with children • Know how to prepare food with children • Incorporate child-friendly tasks into meal preparation
Gardening	• Describe benefits of gardening • Identify materials necessary to start a garden • Plant a simple indoor herb garden
Menu and meal planning	• Recognize importance of planning meals ahead of time • Learn steps to effective meal planning • Review menu and enhance for nutritional balance • Identify food buying strategies to save time and money
Picky eaters, food allergies, and aversions	• Understand differences between picky eater, allergies, and aversion • Learn signs of food allergies • Identify ways to encourage picky eaters
Understanding nutrition facts and how to read a label	• Learn components of a nutrition label • Decipher nutrition fact labels from foods in their home • Learn how to purchase foods for optimal nutrient density

**Table 3:** Happy children’s environmental health intervention core and elective modules and learning objectives

Modules	Provider learning objectives
Core modules	
SMART goals (common with nutrition intervention)	• Describe specific, measurable, attainable, relevant, and timely (SMART) goals • Practice writing a SMART goal • Develop a SMART goal for the family child care home (FCCH) for each subsequent module
Children’s environmental health 101	• Describe why children are more vulnerable to environmental exposures • Understand importance of reducing children’s environmental exposures • Identify potential environmental exposures in FCCHs
Integrated pest management 101	• Discuss current pesticide use in FCCH • Understand concepts of pesticide residue • Understand integrated pest management and less-toxic pest control methods
Staff behaviors: leading the way for safer environments	• Recognize specific behaviors that can reduce environmental exposures • Discuss ways to involve children in actions to reduce potential exposures
Elective Modules	
Green cleaning: sanitizing safely and effectively	• Understand difference between cleaning, sanitizing, and disinfecting • Identify safer cleaning products and practices • Learn ways to reduce exposures to chemicals in cleaning products
Safer toys and art supplies	• Understand importance of choosing safer plastics and art supplies • Recognize third-party certified art supplies • Recognize plastics to avoid
How to read a pesticide label	• Recognize whether a pesticide is legal for use • Understand different sections of a pesticide label and their meaning • Describe importance of using least-toxic pesticides
Steps to pest-free indoor environment	• Recognize situations inside the home that can attract pests • Identify simple and inexpensive methods to prevent, control, and manage indoor pests • Describe importance of using least toxic pesticides indoors
Steps to pest-free outdoor environment	• Recognize situations outside the home that can attract pests • Identify simple and inexpensive methods to prevent, control, and manage outdoor pests • Describe importance of using least toxic pesticides outdoors
Reducing asthma triggers	• Identify triggers of asthma in the indoor environment • Discuss strategies for reducing asthma triggers • Utilize community resources for asthma
Lead safety	• Understand sources and dangers of lead exposures • Identify simple steps to reduce lead exposure • Utilize community resources to further prevent lead exposure
Safer furnishing and napping	• Understand importance of choosing nap equipment and furniture that do not contain harmful chemicals • Recognize toxic products to avoid • Identify simple ways to clean to reduce chemicals and toxins

The two individual educational training sessions, approximately 90 min in length, are conducted by trained interventionists. Interventionists are trained graduate student researchers in the Nutritional Sciences Department of the College of Allied Health and the Department of Health Promotion Sciences in the College of Public Health. Interventionists work with a single FCCH provider at a location of the provider’s choice, typically the provider’s home. The intervention curriculum for these sessions is comprised of multiple modules. The first individual session consists of four modules. The curriculum created for the second individual session offers eight elective modules, from which participants select three.

Thus, during the first two individual sessions, participants complete seven modules: the four core modules and three elective modules. Module activities are designed to increase the self-efficacy of providers. For example, in order to increase self-efficacy for serving correct portion sizes, providers explore a variety of food models and learn appropriate portion sizes to serve in their FCCH. At the end of each module, participants discuss and set specific, measurable, attainable, realistic, and time-sensitive (SMART) goals. Between the first and second sessions, interventionists conduct a check-in telephone call with participants to discuss progress on SMART goals set during the first session and to troubleshoot any challenges. The third and final component of the intervention curriculum is a 3-h group class that is conducted on campus for a small group of participants, scheduled after the baseline visits.

#### The Happy nutrition pilot intervention

The main goals of the nutrition intervention are to: 1) include fruits and vegetables in snacks; 2) eliminate juice; 3) include vegetable subgroup colors throughout the week; 4) serve meals in a family style; and 5) include two servings of whole grains per day. This intervention is timely, as the CACFP guidelines changed substantially in 2017 after nearly 50 years of the same policy [21].

The Academy of Nutrition and Dietetics [74], Academy of Pediatrics, American Public Health Association, and National Resource Center for Health and Safety in Child Care and Early Education [15] have developed benchmarks and standards for nutrition in child care, including not only the nutritional quality of foods served, but also the physical and social environment and behaviors of child care providers. Unfortunately, substantial room for improvement regarding the implementation of these benchmarks exists [75]; providers can improve the dietary intake of young children [76]. Although FCCH providers meet some nutrition recommendations [77] and have more nutrition training than do center-based providers [78], the FCCH nutrition environment may be less healthy than that of the centers [79]. Most FCCHs report that greater emphasis on the unique needs of FCCHs is needed [80]. Two recent systematic reviews that detail aspects of ECE-based interventions to enhance nutrition environments [16, 81] were utilized to shape components of the nutrition intervention.

In addition, we previously conducted a qualitative study of the experiences of 30 FCCH providers with the CACFP before the enforcement of the new meal pattern and their current compliance with recommended best practices [73]. Foods served were determined from menu analyses [73]. This formative work revealed that, while providers are generally in favor of the CACFP, many were unaware of its new rules and best practices. FCCHs in this previous work had areas that needed improvement to continue compliance with the enhanced meal patterns and to meet optional best practices. Identified areas of improvement included appropriate portion sizes, understanding of requirements and best practices and their rationale, reducing sugar, incorporating seasonal and local produce, utilizing family-style meal service, and serving fruits and vegetables as snacks.

Based on the collective findings from our theoretical framework, previous literature, and our formative work, the individual and group sessions were created. The nutrition group class is a cooking class that repeats key project goals and material in addition to hands-on experience with best practices, food preparation tips, sensory experience, and tasting. Recipe preparation and tasting specific to feeding young children is provided. All module content was compared with the Oklahoma Core Competencies for Early Childhood Practitioners [54], the Quality Rating Improvement System Reaching for the Stars Criteria [56], and the Child Development Associate content areas [55]. This crosswalk information on the comprehensive description of the module and theoretical constructs is available upon request from the corresponding author.

#### Details of the Happy environmental health pilot intervention

The main goals of the environmental health intervention are to increase: 1) the knowledge of providers on environmental health; 2) the awareness of providers of potential environmental risks and health consequences in FCCH environments; 3) the knowledge and self-efficacy of providers for risk reduction strategies, such as IPM and green cleaning; and 4) the extent to which providers adopt and carry out behaviors, such as IPM and green cleaning, to make their FCCHs healthier for children and staff.

The environmental health training modules aim to increase the environmental health literacy of FCCH providers, and support practices and behaviors that reduce the exposure of children to chemicals and other toxins found in the FCCH environment. While there have been few studies in FCCHs, previous studies in schools and ECE environments have found levels of chemical residues on surfaces, chemical dusts, allergens, and other contaminants, including known carcinogens and endocrine disruptors, that pose a risk to the health of children in ECE environments [39, 82]. A national environmental health survey of child care centers in the United States found that 75% of child care centers reported at least one pesticide application in the previous year [40].

Environmental health intervention modules were informed by previous research regarding environmental health in ECE centers and FCCH settings, including environmental health interventions conducted with center and FCCH ECE providers [82, 83] in California, where policies are in place to protect children’s environmental health in ECE and schools more broadly. Evidence from these studies and exposure research indicates that strategies such as implementing IPM and green cleaning, proper hand washing behaviors, shoe-free environments, and education for providers as consumers on how to purchase safer and less toxic products are likely to reduce the levels of chemicals and other toxins found in the ECE environments [40–42, 44, 46, 47, 84, 85]. While there have been few FCCH-specific environmental health interventions conducted to date, one California intervention [46] showed that a nurse-led IPM education and consultation intervention yielded significant increases in the IPM knowledge and IPM practices of FCCH providers, and a 90% reduction in the prevalence of pests [46].

The 3-h small group class developed for the environmental health intervention includes further discussion of children’s environment health, IPM, and green cleaning practices. Hands-on activities, such as learning to use a third-party app that can be used to scan cleaning products and identify their relative safety, and making a less toxic all-purpose cleaner for their FCCH, are also included. Participants share their successes and challenges in making changes to their FCCH practices, discuss ideas for making Oklahoma FCCH environments healthier for children, and explore how providers can promote these approaches in their business models. All module content was compared with the Oklahoma Core Competencies for Early Childhood Practitioners [54], the Quality Rating Improvement System Reaching for the Stars Criteria [56], and the Child Development Associate content areas [55]. This crosswalk information on the comprehensive description of the module and theoretical constructs is available upon request from the corresponding author.

### Fidelity evaluation strategies

JaKa et al. [86] and Hoffmann et al. [87] explain that greater detail in intervention reporting is needed for replication. Consistent with the modified version of the National Institutes of Health (NIH) Treatment Fidelity Framework [88], we have details regarding the treatment, training, and intervention delivery for both interventions. These details are described in Table 4. Intervention evaluation follows the RE-AIM framework and includes assessment of the intervention reach, effectiveness, adoption, implementation, and maintenance [89, 90]. Field fidelity checks are conducted by study principal investigators throughout and include interventionist adherence (extent to which practice conforms to the intervention protocol) and competence (skillfulness in the delivery, including interpersonal skills) [91].

**Table 4:** Primary and secondary outcome and fidelity measures for Happy interventions

Intervention	Primary outcome measures	Secondary outcome measures	Fidelity measures
Nutrition technical assistance	• Self-reported nutrition practices • Observed nutrition practices • Observed dietary intake of children • Observed Child and Adult Care Food Program (CACFP) compliance	• Nutrition self-efficacy • Nutrition knowledge • CACFP knowledge • Staff nutrition behaviors • Menu CACFP compliance • Meal service style	• Proportion of participants who complete intervention • Interventionist adherence to intervention curriculum • Interventionist competence in delivering curriculum • Participant satisfaction and overall intervention experience
Children’s environmental health technical assistance	• Self-reported cleaning practices • Self-reported pest-control practices • Observed family child care home (FCCH) environment • Observed chemicals in FCCH	• Self-reported children’s environmental health literacy and knowledge • Self-efficacy for integrated pest management • Self-efficacy for less toxic cleaning practices	

### Outcome evaluation

As a part of the intervention, the FCCH providers will participate in baseline, 3-month, and 12-month follow-up measures (Fig. 1). These measures center on three levels: the individual child (dietary observation), the provider (self-reported measures), and the environment (environmental observation). All measures are collected at two unannounced observation visits, at least 48 h apart, for each time point. Providers are aware of the month of the observation visits, but not the actual days. They can provide up to six blackout dates during which they will not be visited. Table 4 lists the primary and secondary outcomes for both interventions. Detailed data collection tool information is available upon request from the corresponding author.

### Economic evaluation

We are conducting economic evaluations of both interventions. While very little is known about the cost of conducting obesity prevention and environmental health interventions in ECE settings, empirical data are nonexistent for FCCHs [92]. Our evaluation will provide the elements of a cost-effectiveness analysis in which the costs of the intervention (preparation and implementation phases) will be addressed and compared with likely gains in children’s health (e.g., average per-child improvement in body mass index values) estimated from available empirical data on obesity prevention programs and similar environmental health interventions. Resource allocation will be reported using the implementation costs approach since the intervention is at the level of the FCCH provider. This approach will assess the relationship between costs and potential benefits of intervention, and inform replication and scale-up.

Monetary costs of the interventions will be defined as the dollar amount spent on development and implementation. One-time costs, such as project planning and design, will not be included as they would not be incurred subsequently. Major cost categories will include: personnel (salaries, benefits); equipment and materials; travel costs to and from the FCCH; administrative overheads; and other costs related to participant recruitment and incentives. Because participants will keep all intervention materials and equipment after implementation, and the intervention will largely be conducted in their homes, indirect costs to participant are expected to be minimal and will not be considered in the calculations. Using a societal perspective [93], discounted direct and indirect costs over the 3 years of the study will be reported. Following the US Office of Management and Budget’s recommendation, a Marginal Excess Burden will be included in the direct cost calculations [94]. The outcome benchmarks against which we will compare the intervention costs will be specific for the study age group (i.e., preschool children), and will include only those that accounted for significant changes in body mass index [95]. Parameters for the environmental health intervention, such as asthma and respiratory health burdens, will be assessed. Multiple effectiveness estimates will be used to show a range of cost-effectiveness ratios, with the cost per estimated unit of body mass index reduction, in the case of the nutrition intervention, used as the final unit of analysis; similar comparisons will be evaluated with appropriate metrics for the environmental health intervention.

### Sample size determination and power calculation

Sample size calculations were driven by the assumed effect of the nutrition intervention on nutritional measures. The target size of 46 providers provides 80% power to detect a difference between the nutrition intervention and attention control group of 40% adherence for the control versus 80% adherence among the nutrition intervention for a targeted behavior, such as observed CACFP Compliance as an endpoint, assuming a two-sided 0.05 alpha level. Estimates of adherence to dietary guidelines within the control group were derived from the Early Childhood Longitudinal Study among 4-year-old children who attend CACFP-participating center-based ECE [23]. For continuous measures, the targeted sample size will result in 80% power to detect a difference in means, such as the mean CACFP best-practice knowledge score, that is 85% as large as the standard deviation; a “large” effect in the language of Cohen (standardized effect size of 0.8 is a large effect), assuming a two-sided 0.05 alpha level [96]. For child-level measures, assuming that there are two to three eligible children at each center on the observation date, the target sample size of 46 FCCHs and 115 children will result in greater than 80% power to detect a difference in intake of a particular food type, based on the child-level food consumption as described above, between the intervention and control groups of 33% in the control group versus 65% in the intervention group, assuming a two-sided 0.05 alpha level and a within-center correlation of 0.3. An adjusted target sample size of 52 (26 per group) will be used to account for an assumed 10% attrition rate, resulting in an estimated 130 children nested within the FCCH. Sample size calculations were conducted using PASS software [97, 98]. A similar sample size justification can be made for the environmental outcome measures in which the effect sizes are of the same magnitude as assumed for the nutritional intervention.

### Data management and monitoring

Data will be stored in REDCap, and quality control and data checking will occur for a random 10% of participants. If errors are identified, an additional 10% will be reviewed for quality. Data are stored in secured and password-protected servers. Details on data management can be requested from the corresponding author. Given the low-risk behavioral intervention, no Data Safety Committee was created aside from the University Institutional Review Board. Any adverse events, unintended effects, protocol modification, or deviations will be reported to the University as needed. An annual independent review is conducted by the University.

### Data analysis plan

The unit of randomization for data analyses will be the FCCH provider; all child-level outcomes will be analyzed as per the provider-level randomized assignment. An intention-to-treat paradigm [99] will be followed in which data from all eligible participants are analyzed according to randomized intervention assignment, regardless of adherence. A secondary, per-protocol analysis will be performed in which data from providers who attended at least two of the three sessions will be included [100]. Baseline sociodemographic characteristics will be summarized after stratifying by intervention assignment. Comparisons in outcomes will be made between intervention groups using generalized linear mixed models to account for the correlation among measures made on the same provider over time and measures made on children nested within FCCHs [101]. The FCCH and child will be modeled as random effects, with time modeled as a fixed factor. A log-binomial model [102] will be fitted for dichotomous outcomes to measure the prevalence proportion ratio for outcomes, such as serving a specific food type, and a linear model will be fitted for continuous outcomes to estimate the difference in means between groups, such as mean knowledge and self-efficacy scores. Time by intervention interactions will be used to estimate the effect of the interventions on outcomes over time. Nonlinear time trends will be considered and modeled using categorical time variable coding. Baseline assessments of the outcome measures will be included in the model as possible confounding factors [103]. Comparisons between the intervention and attention comparison groups will be made after stratifying by follow-up time point if a significant time by intervention effect is detected. Missing data will be imputed using a multiple imputation technique based on regression modeling as programmed in SAS [104, 105] if necessary.

### Dissemination policy

Scientific dissemination of this trial includes the protocol and methods paper in addition to baseline and follow-up outcome papers for each of the two interventions. Presentation at scientific conferences relevant to nutrition, environmental health, and education will also occur. General summary and infographics will be developed for dissemination to state service partners, nonprofit organizations working with early childhood, and community members. These will be disseminated at meetings, workshops, and conferences at which community members participate.

## Discussion

### Innovation

This protocol provides an overview of a study with a novel research question: will low-cost educational and supportive interventions for FCCH providers result in healthier FCCHs, in terms of nutrition and children’s environmental health? The study that we describe in this manuscript expands ECE research to include the unique FCCH environment and responds to directives from the NAM and others to increase ECE research in environmental and policy interventions [10]. Most obesity prevention interventions conducted to date have focused on center-based ECE [57], which omits 1 million children (26% of all ECE attendance) nationwide who attend FCCHs. While many of these interventions have been successful [57], the structure and dynamics of FCCHs preclude the direct translation from center to FCCHs. Our study examines the effect of an environmental intervention to enhance compliance with CACFP guidelines, which changed in 2017 and have not been previously examined. These outcomes address another NAM research challenge regarding FCCH nutrition [19].

Similarly, our study maximizes opportunity for learning by employing a matched attention comparison intervention trial, in which we develop and test an environmental health intervention with the same study population. Simultaneously testing two interventions is economically advantageous and provides greater participant and ethical engagement, as all research participants are randomized to an intervention with equal contact. This approach will advance understanding of the physical environments and potential environmental health concerns of FCCHs, of which relatively little is known, and also provide much needed evidence about the efficacy of a low-cost environmental health intervention, particularly in FCCHs outside of California where most research to date has been conducted. Furthermore, the interdisciplinary nature of our study team enhances our knowledge of a broader range of children’s health issues and potential intervention strategies in distinct, yet inter-related health areas. Such an approach not only strengthens our study and understanding of ECE environments, but may also move us toward future integrated intervention approaches that promote cohesive FCCH environments and consider the whole environment and whole child.

Including the evaluation of intervention costs in the Happy study is important to advance understanding and provide essential information for state agencies and legislators making funding decisions essential for long-term intervention sustainability, as well as scale-up and replication. Lessons learned and findings of the Happy study will likely be useful to other FCCH networks, in Oklahoma and other states, who increasingly are interested in promoting healthier FCCH environments and children’s health. In the next phase of the Happy study, we plan to conduct an effectiveness trial of the two interventions in FCCHs located in rural counties of Oklahoma. This effectiveness trial will provide further evidence for our interventions, their feasibility, and outcomes.

### Challenges and limitations

No study is without challenges and limitations, and a discussion of those associated with this study is warranted. First, although we will be using validated study instruments and measures, several outcomes include self-report measures that are subject to social desirability bias. Some of the measures for the environmental health intervention study were created for our study, since the limited amount of research in this area to date precludes the use of previously validated measures. Similarly, FCCH providers may alter their behaviors or foods served on the days of observation. As the dates of observation are unannounced, this is unlikely but still possible. Since participation is voluntary, it is possible that providers who could substantially benefit have not enrolled; thus, findings may not be generalizable to all FCCH providers. Furthermore, contextual differences related to the launching and enforcement of the new CACFP rules and best practices may affect the implementation and impact of the two study interventions. The impact of these differences remains to be evaluated.

Since recruitment and informed consent procedures require that FCCH providers be told they have a 50/50 chance of being assigned to either the nutrition or environmental health intervention, some providers may be disappointed with their randomization assignment. While this phenomenon is not unique to this study, a potentially unique lesson that we have learned to date is that FCCHs in our area are actively involved in closed social media groups for FCCH providers, and frequently discuss issues relevant to their FCCHs, including our study. For example, some providers who are participants in one of our intervention arms have communicated with other providers participating in the other intervention arm, and requested that they share the information and materials they received. While this type of cross-talk about treatment assignments is inevitable in a small community and, in many ways, expresses the enthusiasm that our participants have for our study and the care they provide, we had not anticipated that this professional community would be this connected, at least in the Oklahoma City area. Future research, including interventions with FCCHs, may want to account for this type of interaction and possibly leverage it for intervention and information dissemination with FCCH providers.

Recruitment of participants can be a challenge in any study. Partnership with the Oklahoma Child Care Resource and Referral agency and CACFP sponsors has been essential in recruitment efforts. Initial recruitment efforts included attendance at training events coordinated by CACFP sponsoring organizations, which FCCH providers are required to attend. Providers were given a brief introduction to the study and could contact the research team if interested. We also attended several local FCCH professional membership-based organizations. This effort was effective early on, but we shifted to a more active recruitment method to reach those who may not have had an initial interest. We partnered with the sponsoring organizations, directly called the FCCH providers, and invited them to participate. This was an effective strategy, although it has resulted in more providers who screen in, but do not complete or even start the intervention. While this is not entirely unexpected with the more active recruitment strategy, we have observed greater reach when employing this method.

## Conclusions and impact

Early prevention of obesity and potentially hazardous environmental exposures in environments where young children spend substantial time, such as ECE settings, is essential for safeguarding children’s health development and wellness across the life course. The Happy study aims respond to ECE and CACFP research priorities stated by the NAM [10, 19], as well as the critical need to address early childhood obesity, asthma, and other associated health outcomes in Oklahoma’s children, particularly under-served and low-income populations. Successful completion of this study will provide critical information about the quality of nutrition and broader physical environments faced by young children in FCCHs, as well as much needed evidence about the efficacy of two low-cost, community-based interventions to protect and promote children’s health in ECE environments.

### Trial status

Enrollment and intervention are complete. Follow-up measures are underway.

## Additional files

Additional file 1 SPIRIT 2013 checklist: recommended items to address in a clinical trial protocol and related documents. (DOC 119 kb)

Additional file 2: Appendix A: World Health Organization Trial Registration Data Set. Appendix B: Consent form. (DOCX 366 kb)

## Data Availability

Data sharing is not applicable to this article, as no datasets were generated or analyzed for this manuscript. SBS and ALS will have access to the final trial dataset and data use agreements can be established with the University of Oklahoma Health Sciences Center and the investigative team. Authorship criteria align with the ICMJE guidelines.

## Abbreviations

CACFP: Child and Adult Care Food Program
ECE: Early care and education
FCCH: Family child care home
IPM: Integrated pest management
NAM: National Academy of Medicine
NIH: National Institutes of Health
RE-AIM: Reach, effectiveness, adoption, implementation, maintenance
SMART: Specific, measurable, attainable, realistic, and time-sensitive goals

## References

[1] National Resource Council, Institute of Medicine (2000). From neurons to neighborhoods: the science of early childhood development.

[2] Fox SE, Levitt P, Nelson CA (2010). How the timing and quality of early experiences influence the development of brain architecture. Child Dev.

[3] Donoghue Elaine A. (2017). Quality Early Education and Child Care From Birth to Kindergarten. Pediatrics.

[4] Early Childhood Program Participation Survey of the National Household Education Surveys Program. 2019. [http://nces.ed.gov/programs/digest/d09/tables/dt09_044.asp].

[5] Redford J, Desrochers D, Mulvaney Hoyer K. The years before school: children's nonparental care arrangements from 2001 to 2012. In: Stats in brief. Washington, DC: US Department of Education, Institute of Education Sciences; 2017.

[6] National Resource Council, Institute of Medicine (2013). Institute of Medicine: US health in international perspective: shorter lives, poorer health. panel on understanding cross-national health differences among high-income countries.

[7] Birch Leann L. (1998). Psychological Influences on the Childhood Diet. The Journal of Nutrition.

[8] Nahikian-Nelms M (1997). Influential factors of caregiver behavior at mealtime: a study of 24 child-care programs. J Am Diet Assoc.

[9] Barnes M. Task Force on Childhood Obesity: White House Task Force on childhood obesity report to the President: solving the problem of childhood obesity within a generation. In: Task force on childhood obesity; 2010. http://www.letsmove.gov/white-house-task-force-childhood-obesity-report-president. Accessed Mar 2016. 10.1089/bfm.2010.9980 20942695

[10] Institute of Medicine (2011). Early childhood obesity prevention policies.

[11] A blueprint for protecting children’s environmental health: an urgent call to action. 2019. [https://cehn.org/wp-content/uploads/2015/11/BluePrint_Final1.pdf].

[12] National Association of County and City Health Officials policy statement 00–12: children’s environmental health. 2019. [https://www.naccho.org/uploads/downloadable-resources/99-12-Childrens-Environmental-Health.pdf].

[13] American Academy of Pediatrics, American Public Health Association, National Resource Center for Health and Safety in Child Care and Early Education: Caring for our children: National health and safety performance standards; Guidelines for early care and education programs (3rd ed.). Elk Grove Village, IL: American Academy of Pediatrics; 2011.

[14] Protecting Children's Environmental Health: a comprehensive framework (policy number: 201710). 2019. [https://www.apha.org/policies-and-advocacy/public-health-policy-statements/policy-database/2018/01/23/protecting-childrens-environmental-health].

[15] American Academy of Pediatrics, American Public Health Association, National Resource Center for Health and Safety in Child Care and Early Education: Preventing Childhood Obesity in Early Care and Education Program: Selected Standards from Caring for Our Children: National Health and Safety Performance Standards; Guidelines for Early Care and Education Programs, 3rd Edition, Second edn: Aurora, CO; 2012.

[16] Sisson SB, Krampe M, Anundson K, Castle S (2016). Obesity prevention interventions in child care: a systematic review. Prev Med Rep.

[17] Research brief #2: Trends in family child care home licensing regulations and policies for 2014. 2019. [http://www.naralicensing.org/2014-cc-licensing-study].

[18] Oklahoma Department of Human Services (2013). Licensing requirements for family child care homes and large child care homes.

[19] Institute of Medicine (2012). Research methods to assess dietary intake and program participation in child day care.

[20] LetsMove: ChildCare. 2019. [https://healthykidshealthyfuture.org/].

[21] Child and Adult Care Food Program. 2019. [http://www.fns.usda.gov/cacfp/meals-and-snacks].

[22] Ritchie LD, Boyle M, Chandran K, Spector P, Whaley SE, James P, Samuels S, Hecht K, Crawford P (2012). Participation in the child and adult care food program is associated with more nutritious foods and beverages in child care. Child Obes.

[23] Korenman S, Abner KS, Kaestner R, Gordon RA (2013). The Child and Adult Care Food Program and the nutrition of preschoolers. Early Child Res Q.

[24] Schwartz MB, Henderson KE, Grode G, Hyary M, Kenney EL, O'Connell M, Middleton AE (2015). Comparing current practice to recommendations for the Child and Adult Care Food Program. Child Obes.

[25] Monsivais P, Kirkpatrick S, Johnson DB (2011). More nutritious food is served in child-care homes receiving higher federal food subsidies. J Am Diet Assoc.

[26] Healthy Eating, Active Play, Screen Time Best Practices. 2019. [http://www.publichealthlawcenter.org/heal/ChildCareMaps.html]

[27] Bouchard MF, Chevrier J, Harley KG, Kogut K, Vedar M, Calderon N, Trujillo C, Johnson C, Bradman A, Barr DB (2011). Prenatal exposure to organophosphate pesticides and IQ in 7-year-old children. Environ Health Perspect.

[28] Eskenazi B, Marks AR, Bradman A, Harley K, Barr DB, Johnson C, Morga N, Jewell NP (2007). Organophosphate pesticide exposure and neurodevelopment in young Mexican-American children. Environ Health Perspect.

[29] Jurewicz J, Hanke W, Johansson C, Lundqvist C, Ceccatelli S, van den Hazel P, Saunders M, Zetterstrom R (2006). Adverse health effects of children's exposure to pesticides: what do we really know and what can be done about it. Acta Paediatr Suppl.

[30] Makri A, Goveia M, Balbus J, Parkin R (2004). Children's susceptibility to chemicals: a review by developmental stage. J Toxicol Environ Health B Crit Rev.

[31] Moya J, Bearer CF, Etzel RA (2004). Children's behavior and physiology and how it affects exposure to environmental contaminants. Pediatrics.

[32] Rauh VA, Garfinkel R, Perera FP, Andrews HF, Hoepner L, Barr DB, Whitehead R, Tang D, Whyatt RW (2006). Impact of prenatal chlorpyrifos exposure on neurodevelopment in the first 3 years of life among inner-city children. Pediatrics.

[33] Zota Ami R, Singla Veena, Adamkiewicz Gary, Mitro Susanna D, Dodson Robin E (2017). Reducing chemical exposures at home: opportunities for action. Journal of Epidemiology and Community Health.

[34] United States Environmental Protection Agency (2002). Child-specific exposure factors handbook.

[35] Bradman A, Castorina R, Gaspar F, Nishioka M, Colon M, Weathers W, Egeghy PP, Maddalena R, Williams J, Jenkins PL (2014). Flame retardant exposures in California early childhood education environments. Chemosphere.

[36] Breysse P, Farr N, Galke W, Lanphear B, Morley R, Bergofsky L (2004). The relationship between housing and health: children at risk. Environ Health Perspect.

[37] Gaspar FW, Castorina R, Maddalena RL, Nishioka MG, McKone TE, Bradman A (2014). Phthalate exposure and risk assessment in California child care facilities. Environ Sci Technol.

[38] Quiros-Alcala L, Wilson S, Witherspoon N, Murray R, Perodin J, Trousdale K, Raspanti G, Sapkota A (2016). Volatile organic compounds and particulate matter in child care facilities in the District of Columbia: results from a pilot study. Environ Res.

[39] Environmental exposures in childhood education environments. Report Prepared for the California Air Resources Board. 2019. [https://www.arb.ca.gov/research/apr/past/08-305.pdf].

[40] Tulve NS, Jones PA, Nishioka MG, Fortmann RC, Croghan CW, Zhou JY, Fraser A, Cavel C, Friedman W (2006). Pesticide measurements from the first national environmental health survey of child care centers using a multi-residue GC/MS analysis method. Environ Sci Technol.

[41] Goveia M, Shaikh N, Windham G, Bembom O, Feldman K, Kreutzer R (2005). Asthma-related environmental practices and asthma awareness in California child care centers. Pediatr Asthma Allergy Immunol.

[42] Morgan MK, Sheldon LS, Croghan CW, Jones PA, Chuang JC, Wilson NK (2007). An observational study of 127 preschool children at their homes and daycare centers in Ohio: environmental pathways to cis- and trans-permethrin exposure. Environ Res.

[43] Alkon A, Kalmar E, Leonard V, Flint ML, Kuo D, Davidson N, Bradman A: Development and evaluation of an integrated pest management toolkit for child care providers. Early Child Res Pract 2012, 14(2).

[44] Bradman A, Dobson C, Leonard V (2010). Pest management and pesticide use in California child care centers. In.

[45] Mir DF, Finkelstein Y, Tulipano GD (2010). Impact of integrated pest management (IPM) training on reducing pesticide exposure in Illinois childcare centers. Neurotoxicology.

[46] Stephens Michelle, Hazard Kimberly, Moser Debra, Cox Dana, Rose Roberta, Alkon Abbey (2017). An Integrated Pest Management Intervention Improves Knowledge, Pest Control, and Practices in Family Child Care Homes. International Journal of Environmental Research and Public Health.

[47] Alkon A, Nouredini S, Swartz A, Sutherland AM, Stephens M, Davidson NA, Rose R (2016). Integrated pest management intervention in child care centers improves knowledge, pest control, and practices. J Pediatr Health Care.

[48] Israel BA, Schulz AJ, Parker EA, Becker AB (1998). Review of community-based research: assessing partnership approaches to improve public health. Annu Rev Public Health.

[49] Minkler M (2005). Community-based research partnerships: challenges and opportunities. J Urban Health.

[50] Faulkner LA, Riley J. In: Sisson SB, editor. OKDHS child care home and capacity and children in subsidized care as of December 2015. Oklahoma City: Oklahoma Department of Human Services; 2016.

[51] Weber J: Tier of FCCH provider reimbursement. In*.* Edited by Sisson SB; 2016.

[52] Referral RFCCRa: FY 2014 Annual Report. In*.*; 2015.

[53] Ostbye T, Mann CM, Vaughn AE, Brouwer RJN, Neelon SEB, Hales D, Bangdiwala SI, Ward DS (2015). The keys to healthy family child care homes intervention: study design and rationale. Contemp Clin Trials.

[54] Oklahoma Department of Human Services. OCCS: Oklahoma core compentencies for early childhood practitioners. Oklahoma City: Oklahoma Department of Human Services and Oklahoma Child Care Services; 2008.

[55] Preschool child development associate subject areas. 2019. [https://www.carecourses.com/PublicPages/Set_CDA_Preschool_Chart.aspx].

[56] Oklahoma Department of Human Services: Reaching for the stars for child care programs quality rating improvement system. In*.*, vol. 2018: Oklahoma Department of Human Services, Child Care Services; 2016.

[57] Sisson Susan B., Krampe Megan, Anundson Katherine, Castle Sherri (2016). Obesity prevention and obesogenic behavior interventions in child care: A systematic review. Preventive Medicine.

[58] Sallis JF, Owen N, Fisher EB. Ecological models of health behavior. In K. Glanz, B. K. Rimer, & K. Viswanath (Eds.), Health behavior and health education: Theory, research, and practice (pp. 465-485). San Francisco: Jossey-Bass; 2008.

[59] Bronfenbrenner U (1994). Ecological models of human development. International encyclopedia of education. vol. 3.

[60] Bronfenbrenner U, Ceci SJ (1994). Nature-nurture reconceptualized in developmental perspective: a bioecological model. Psychol Rev.

[61] Fisher EB, Walker EA, Bostrom A, Fischhoff B, Haire-Joshu D, Johnson SB (2002). Behavioral science research in the prevention of diabetes: status and opportunities. Diabetes Care.

[62] Fielding JE, Teutsch S, Breslow L (2010). A framework for public health in the United States. Public Health Rev.

[63] Friedman DJ, Starfield B (2003). Models of population health: their value for US public health practice, policy, and research. Am J Public Health.

[64] Bronfenbrenner U (1979). The ecology of human development (experiments by nature and design).

[65] Bandura A (1997). Self efficacy: the exercise of control.

[66] Bandura A (1986). Social foundations of thought and action: a social cognitive theory.

[67] Heaney CA, Israel BA, Glanz K (2002). Social networks and social support. Health behavior and heatlh education: theory, research, and practice.

[68] Ryan RM, Deci EL (2000). Self-determination theory and the facilitation of intrinsic motivation, social development, and well-being. Am Psychol.

[69] Committee o Developments in the Science of Learning (2000). Committee on learning research and educational practice, national research council: how people learn: brain, mind, experience, and school: expanded edition.

[70] Sallis JF, Owen N, Glanz K, RB, Lewis FM (2002). Ecological models of health behavior. Health behavior and health education: theory, research, and practice.

[71] Taveras EM, LaPelle N, Gupta RS, Finkelstein JA (2006). Planning for health promotion in low-income preschool child care settings: focus groups of parents and child care providers. Ambul Pediatr.

[72] Copeland KA, Kendeigh CA, Saelens BE, Kalkwarf HJ, Sherman SN (2012). Physical activity in child-care centers: do teachers hold the key to the playground?. Health Educ Res.

[73] Sisson Susan B., Brice Ashley M., Hoffman Leah A., Lazarte-Alcala Naneida, Faulkner-Schnieder LuAnn, Weber Jennifer, Knehans Allen W. (2018). Family Child Care Home Provider Experiences with the Child and Adult Care Food Program. Journal of Nutrition Education and Behavior.

[74] Benjamin Neelon SE, Briley ME (2011). Position of the American Dietetic Association: benchmarks for nutrition in child care. J Am Diet Assoc.

[75] Dev DA, McBride BA, Team SKR (2013). Academy of Nutrition and Dietetics benchmarks for nutrition in child care 2011: are child-care providers across contexts meeting recommendations?. J Acad Nutr Diet.

[76] Ward S, Belanger M, Donovan D, Carrier N (2015). Systematic review of the relationship between childcare educators' practices and preschoolers' physical activity and eating behaviours. Obes Rev.

[77] Trost SG, Messner L, Fitzgerald K, Roths B (2009). Nutrition and physical activity policies and practices in family child care homes. Am J Prev Med.

[78] Kim J, Shim JE, Wiley AR, Kim K, McBride BA (2012). Is there a difference between center and home care providers' training, perceptions, and practices related to obesity prevention?. Matern Child Health J.

[79] Martyniuk OJM, Vanderloo LM, Irwin JD, Burke SM, Tucker P (2016). Comparing the nutrition environment and practices of home- and centre-based child-care facilities. Public Health Nutr.

[80] Briley ME, Buller AC, Roberts-Gray CR, Sparkman A (1989). What is on the menu at the child care center?. J Am Diet Assoc.

[81] Ward DS, Welker E, Choate A, Henderson KE, Lott M, Tovar A, Wilson A, Sallis JF (2017). Strength of obesity prevention interventions in early care and education settings: a systematic review. Prev Med.

[82] Green cleaning, sanitizing, and disinfecting: a toolkit for early care and education. 2019. [http://cerch.berkeley.edu/sites/default/files/green_cleaning_toolkit.pdf].

[83] Integrated pest management: a curriculum for early care and education programs. 2019. [http://wspehsu.ucsf.edu/wp-content/uploads/2015/10/Curriculum_FINAL-12.2010.pdf].

[84] Lu C, Adamkiewicz G, Attfield KR, Kapp M, Spengler JD, Tao L, Xie SH (2013). Household pesticide contamination from indoor pest control applications in urban low-income public housing dwellings: a community-based participatory research. Environ Sci Technol.

[85] Wilson NK, Chuang JC, Lyu C (2001). Levels of persistent organic pollutants in several child day care centers. J Expo Anal Environ Epidemiol.

[86] JaKa MM, Haapala JL, Trapl ES, Kunin-Batson AS, Olson-Bullis BA, Heerman WJ, Berge JM, Moore SM, Matheson D, Sherwood NE (2016). Reporting of treatment fidelity in behavioural paediatric obesity intervention trials: a systematic review. Obes Rev.

[87] Hoffmann TC, Glasziou PP, Boutron I, Milne R, Perera R, Moher D, Altman DG, Barbour V, Macdonald H, Johnston M (2014). Better reporting of interventions: template for intervention description and replication (TIDieR) checklist and guide. BMJ.

[88] Borrelli B, Sepinwall D, Ernst D, Bellg AJ, Czajkowski S, Breger R, DeFrancesco C, Levesque C, Sharp DL, Ogedegbe G (2005). A new tool to assess treatment fidelity and evaluation of treatment fidelity across 10 years of health behavior research. J Consult Clin Psychol.

[89] Glasgow RE, McKay HG, Piette JD, Reynolds KD (2001). The RE-AIM framework for evaluating interventions: what can it tell us about approaches to chronic illness management?. Patient Educ Couns.

[90] Glasgow RE, Vogt TM, Boles SM (1999). Evaluating the public health impact of health promotion interventions: the RE-AIM framework. Am J Public Health.

[91] Breitenstein SM, Fogg L, Garvey C, Hill C, Resnick B, Gross D (2010). Measuring implementation fidelity in a community-based parenting intervention. Nurs Res.

[92] Wright DR, Kenney EL, Giles CM, Long MW, Ward ZJ, Resch SC, Moodie ML, Carter RC, Wang YC, Sacks G (2015). Modeling the cost effectiveness of child care policy changes in the U.S. Am J Prev Med.

[93] Siegel JE, Weinstein MC, Russell LB, Gold MR (1996). Recommendations for reporting cost-effectiveness analyses. Panel on Cost-Effectiveness in Health and Medicine. JAMA.

[94] Office of Management and Budget: Circular A-94 (1992). Guidelines and discount rates for benefit-cost analysis of federal programs.

[95] Wang Y, Wu Y, Wilson R, Bleich S, Cheskin L, Weston C, Showell N, Fawole O, Lau B, Segal J (2013). Childhood obesity prevention programs: comparative effectiveness review and meta-analysis.

[96] Cohen J (1992). A power primer. Psychol Bull.

[97] Donner A, Klar N (2000). Design and analysis of cluster tandomization trials in health research.

[98] PASS 13. 2019. www.ncss.com.

[99] Gupta S (2011). Intention-to-treat concept: a review. Perspect Clin Res.

[100] Sainani KL (2010). Making sense of intention-to-treat. Phys Med Rehabil.

[101] McCulloch CE, Neuhaus JM. Generalized linear mixed models. Wiley; 2013.

[102] Petersen MR, Deddens JA (2008). A comparison of two methods for estimating prevalence ratios. BMC Med Res Methodol.

[103] Maldonado G, Greenland S (1993). Simulation study of confounder-selection strategies. Am J Epidemiol.

[104] Schafer JL (1999). Multiple imputation: a primer. Stat Methods Med Res.

[105] Yuan YC (2010). Multiple imputation for missing data: concepts and new development (version 9.0), vol. 49.

