# Supplementary material for: Interventions to promote healthy environments in family child care homes in Oklahoma—Happy Healthy Homes: study protocol for a randomized controlled trial
Source: Trials. 2019 Aug 30;20:541. doi: 10.1186/s13063-019-3616-9 (PMC6716934; doi:10.1186/s13063-019-3616-9)
Supplement: Supplementary file 2 — Appendix A: World Health Organization Trial Registration Data Set. Appendix B: Consent form. (DOCX 366 kb) [file 13063_2019_3616_MOESM2_ESM.docx]

Appendix A: World Health Organization Trial Registration Data Set

| Primary registry and trial identifying number | NCT03560050 |
| --- | --- |
| Date of registration in primary registry | May 23, 2018 |
| Secondary identifying numbers | none |
| Source(s) of monetary or material support | United States Department of Agriculture |
| Primary sponsor | United States Department of Agriculture |
| Secondary sponsor(s) | none |
| Contact for public queries | Susan B Sisson, PhD, RDN,CHES  University of Oklahoma Health Sciences Center, Oklahoma City, OK, USA |
| Contact for scientific queries | Susan B Sisson, PhD, RDN,CHES  University of Oklahoma Health Sciences Center, Oklahoma City, OK, USA |
| Public title | Technical Assistance for Child and Adult Care Food Program in Family Child Care Home |
| Scientific title | Compliance and Technical Assistance for Child and Adult Care Food Program in Family Child Care Homes |
| Countries of recruitment | United States of America |
| Health condition(s) or problem(s) studied | - Nutrition Poor - Health Behavior |
| Intervention(s) | Behavioral training |
| Key inclusion and exclusion criteria | - Family child care home providers within 60 minutes of Oklahoma City who participate in the Child and Adults Care Food Program |
| Study type | Allocation: Randomized Intervention Model: Parallel Assignment Intervention Model Description:  Randomized trial where family child care home providers (the participant) are randomized to either nutrition or environmental health technical assistance intervention. Interventions are matched for time and contact  Masking: None (Open Label) Primary Purpose: Prevention |
| Date of first enrollment | October 1, 2018 |
| Target sample size | 52 |
| Recruitment status | recruiting |
| Primary outcome(s) | Change in self-reported nutrition and environmental health practices |
| Key secondary outcomes | Change in nutrition environment; Change in compliance of menu and meal with Child and Adult Care Food Program requirements; Change in children's dietary intake; Change in Environmental health observation |

Appendix B: Consent
